# Light People: Professor John Dudley spoke about supercontinuum generation and International Day of Light

**DOI:** 10.1038/s41377-021-00653-z

**Published:** 2021-10-07

**Authors:** Chenzi Guo

**Affiliations:** Light Publishing Group, Changchun Institute of Optics, Fine Mechanics and Physics, Chinese Academy of Sciences, 3888 Dong Nan Hu Road, Changchun, 130033 China

**Keywords:** Nonlinear optics, Ultrafast photonics

## Abstract

The extreme spectral broadening of light in supercontinuum generation (SCG) is considered by many as the ultimate legacy of nonlinear optics. In this interview, *Light: Science & Applications* invited John Dudley [see the “Short Bio” section]—pioneer of supercontinuum generation, rogue waves, and ultrafast lasers—to share insight on how supercontinuum generation have evolved over the past decades and where it is heading. Also as the Steering Chair of UNESCO’s International Day of Light & International Year of Light (IDL & IYL), John is asked to share his comments on how light may influence post-pandemic World.

**Fig. 1:**
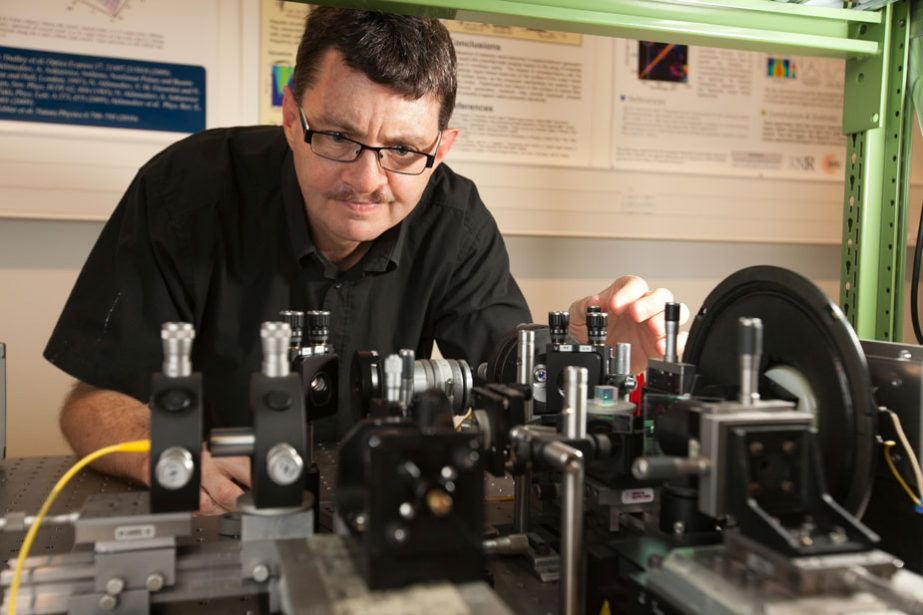
Credit to: Ludovic Godard, UFC.

**Short Bio:** Dr., Prof. John Dudley received his Ph.D. from the University of Auckland and is currently a Professor at the University of Franche-Comté in Besançon, France. He is a Fellow of OSA, SPIE, IEEE, EOS, and an Honorary Fellow of the Royal Society of New Zealand Te Aparangi.

Dr. Dudley’s research covers a wide range of topics in ultrafast and nonlinear optics. In addition to early work in source development, he pioneered the use of advanced measurement techniques to characterize complex pulse propagation in nonlinear fiber optics, and contributed especially to the development of a clear understanding of the physics of fiber supercontinuum generation.

He has published over 500 contributions in journals & conference proceedings and delivered over 120 invited talks at major conferences. He served as the President of the European Physical Society from 2013 to 2015, and has served on a number of editorial boards and conference committees.

Dr. Dudley is also known for initiating the International Year of Light and chairing the Steering Committee of IYL and IDL. He is a keen educator in teaching undergraduate lectures and specialised graduate courses with both English and French.

Dr. Dudley is the recipient of the IXCore Fondation pour La Recherche Prize, the Grand Prix de l’Electronique Général FERRIÉ of the Société des Electriciens et Electroniciens, the Médaille d’Argent of the national French research agency CNRS, the SPIE President’s Award, the OSA Hopkins Leadership Award, the IOP President’s Medal and the APS Dwight Nicholson Medal for Outreach. In 2019 he was awarded the Harold E. Edgerton Award of SPIE recognizing his contributions to ultrashort pulse measurements in nonlinear fibre optics. In 2020 he was awarded the R. W. Wood Prize of OSA recognizing his contributions to explaining and interpreting the physics of fibre supercontinuum generation, and was elected an Honorary Fellow of the Royal Society of New Zealand Te Aparangi.

**Q1:** **What is the present situation with supercontinuum generation? What are the current major challenges?**

A1: The supercontinuum is a remarkable light source where multiple nonlinear processes in an optical waveguide combine to transform a narrowband input into a broadband spectrum, capable of spanning from the ultraviolet to the infrared. The way in which the frequency conversion processes occur depends critically on the dispersion engineering of the waveguide, so extensive work is now using advances in waveguide engineering and fabrication to design supercontinuum spectra optimized at specific wavelengths for specific applications.

The overall physics is now well understood, but a number of new challenges are emerging in trying to understand how cascading different waveguides can be used to sequentially seed different nonlinear processes. A great deal of work continues to studying supercontinuum noise in order to determine the fundamental limits to coherence, with continued interest in the development of frequency comb technology. And researchers are also moving beyond the single mode fiber platform to consider the additional degrees of freedom afforded from control of multimode interactions.

**Q2: Supercontinuum generation has been demonstrated spanning the ultraviolet, visible and near infrared range. In the future, how broadband supercontinuum can be? What are the limiting factors?**

A2: The obvious limits to achievable bandwidth are the factors such as material attenuation and the amount of power one can couple into the system before reaching the material damage threshold. A key advance that has managed to overcome these limits has been spectral broadening using soliton dynamics in hollow core fibres. This has actually been very significant in allowing access to the important ultraviolet spectral range approaching the boundary of attoscience. Results in the four-octave range have been reported, and these are really remarkable. In this regime, other limiting factors associated with plasma and related dynamics may also begin to play a role, which will evoke fascinating physics and inspire a lot of work in this field of gas-based nonlinear optics.

**Q3: Photonic crystal fibers enabled supercontinuum generation without high power lasers. What are the other options for generating supercontinuum without high power lasers?**

A3: It depends on your application, because some applications will always require high spectral density to achieve good signal to noise and in such cases, the pump source will likely also need to satisfy minimum power requirements. But in general, the conversion efficiency into particular wavelength bands depends critically on the waveguide design and dispersion characteristics, and for any given pump, the key is to optimize the dispersion tailoring to favor the generation of spectral components where they are desired. The development of compact integrated waveguides with novel materials and precise dispersion engineering is proving especially effective here, and many impressive results are being reported.

**Q4: How do Rogue waves affect supercontinuum?**

A4: Optical rogue waves are high intensity pulses of light that appear in a supercontinuum as a result of noise-sensitive soliton dynamics. Rogue waves in a supercontinuum are usually associated with shifting the bandwidth towards longer wavelengths, and although they are necessarily associated with shot-to-shot instability, for many applications this is not important when high repetition rate sources are used. A potential deleterious effect can also occur, however, if the rogue wave intensity at some point in the waveguide exceeds the damage threshold. One of the important open questions here is to try to determine how to exploit rogue wave dynamics in a controlled way to develop practical methods for supercontinuum spectral engineering.

**Q5: What changes will machine learning bring to supercontinuum?**

A5: Machine learning is already becoming part of the standard toolbox used in nonlinear optics. Optimization algorithms have already been used in experiments to modify initial conditions to generate supercontinnum with desired spectral properties, and this approach—using genetic algorithms and/or neural networks—is likely to become widespread. There is also tremendous potential in the real-time control of pump laser systems used in experiments, and from a theoretical perspective, neural networks have already proved to be very powerful in identifying patterns and correlations in supercontinuum dynamics.

**Q6: Supercontinuum has found significant applications in spectroscopy and microscopy, what are the other possibilities for supercontinuum that has not been well aware of yet?**

A6: The supercontinuum is a versatile and flexible light source and has potential applications anywhere light is used. Whether the emphasis is on generating light within a particular wavelength range or over a broad bandwidth, there is usually a supercontinuum source that provides what is needed. The issue here of course is compactness and cost! Amongst some of the emerging applications that are attracting wide interest are the applications of supercontinuum in the mid-infrared—in extending the bandwidths of fiber laser sources, and in developing frequency combs with very low amplitude and phase noise.

**Q7: More broadly, what are the most promising directions in nonlinear optics and ultrafast lasers?**

A7: What a question! Probably every researcher in the field will have their own favorite answer, but with my own interest in the field of more basic research, I am really interested in understanding how the techniques of machine learning can help us gain new insights into light–matter interactions. In addition to optimization approaches, machine learning brings in very advanced techniques for data analysis, and new ways to attempt to infer the underlying physics from data-driven discovery. I think there are some fascinating years ahead to see where this will take us.

**Q8: We first met in 2017 at the IDL meeting at CLEO Munich, where light, its applications and how IDL shall improve the impact of light were extensively discussed. Looking back the past few years, can you name a few things that IYL & IDL has brought changes to? Also, could you share insight on how light may influence the post-pandemic world and how our researchers can utilize the interplay?**

A8: IYL and IDL have succeeded beyond anything we initially hoped for. Concretely, since 2015 they have led to over 15000 outreach events that have reached millions of people, all focused around light and photonics. On a societal level, I think we have raised awareness of the importance of light science for sustainable development, and many of the themes we have promoted have found their way into government and political statements. For example, in 2021 we focused on a campaign aiming to promote trust in science (https://www.lightday.org/trust-science), and this was adopted by UNESCO and stressed explicitly by their Director General of UNESCO in an important public message (https://unesdoc.unesco.org/ark:/48223/pf0000377406_eng.locale=en) in May. The central role played by science in society has been shown very clearly over the last 18 months, where we have clearly seen the importance of long-term investment in basic research and technology - from the science of vaccines to the infrastructure photonic-based communications networks. I think it is essential that researchers learn how to argue for more long-term investment in science. Our future depends on it.

**Fig. 2:**
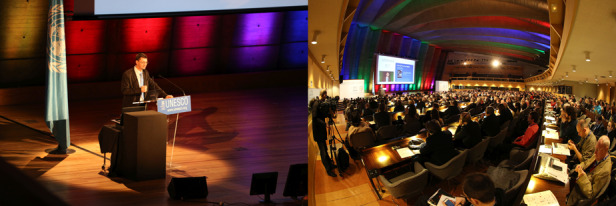
Prof. Dudley spoke at the Opening Ceremony of IYL. Credit to: Dan Curticapean.

**Fig. 3:**
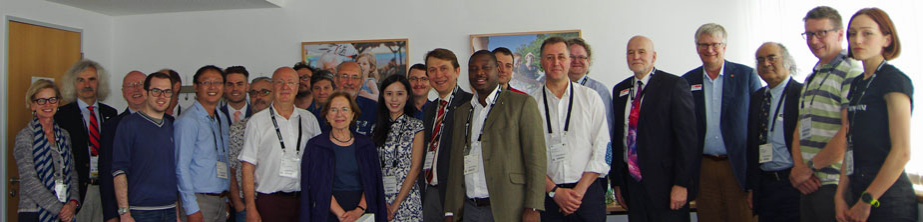
2017 IDL Steering Committee in Munich.

**Fig. 4:**
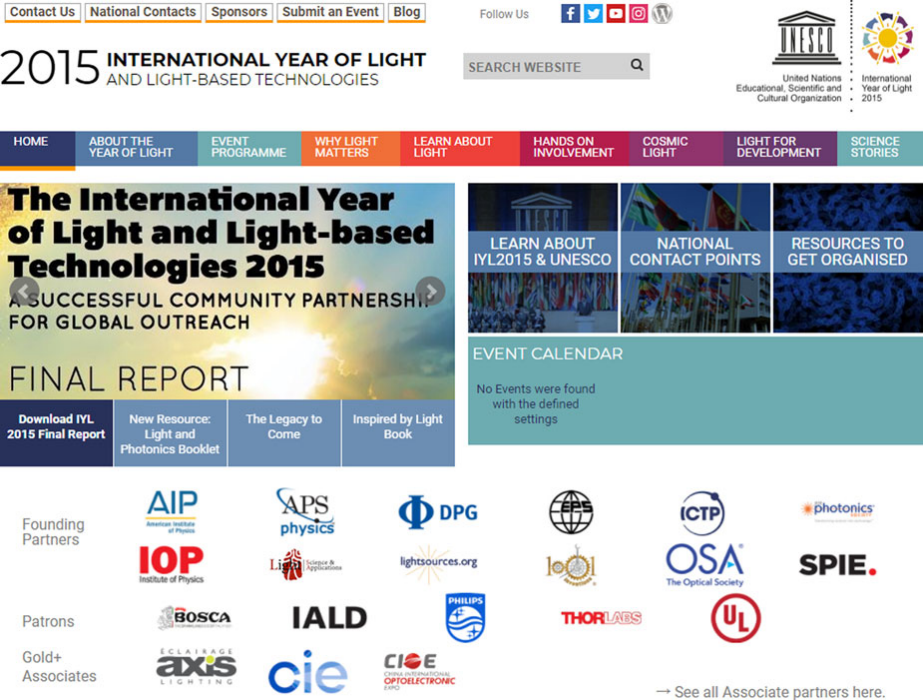
Homepage of IYL. Credit to: IYL.

**Fig. 5:**
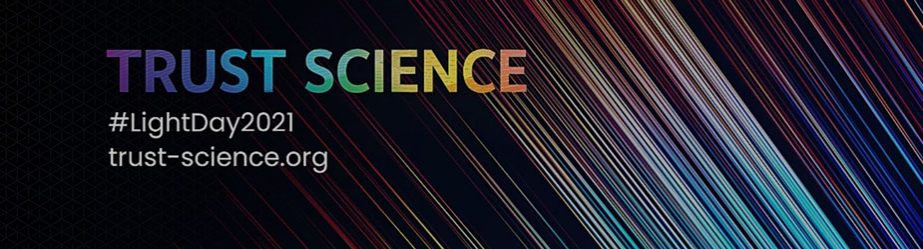
Trust Science Event Banner. Credit to: IDL.

**Q9: You have always been a dedicated educator, and quite creative in live-science lectures. What is the difference between conveying information to students and the general public, what different moves do you usually take?**

A9: I have always found that independent of the audience, the essential thing is to ensure that you try to first explain the concepts in very general terms, and that these are linked quickly with concrete real-world applications. That is, you start by asking the question “Why is this interesting and important”, and you give a short answer to this question before getting into detail. Hopefully, this attracts people’s interest and inspires them to want to learn more about the topic. For both the general public and students, I also try to use simple experiments to illustrate the point, and we are lucky in optics that we have such a visual subject. Of course the big difference when teaching students at university is that we extend conceptual explanations with mathematical modelling, trying to explain what we see quantitatively and hopefully predicting effects we hadn’t expected. When this forces us to change our conceptual picture, then we have really succeeded!

**Fig. 6:**
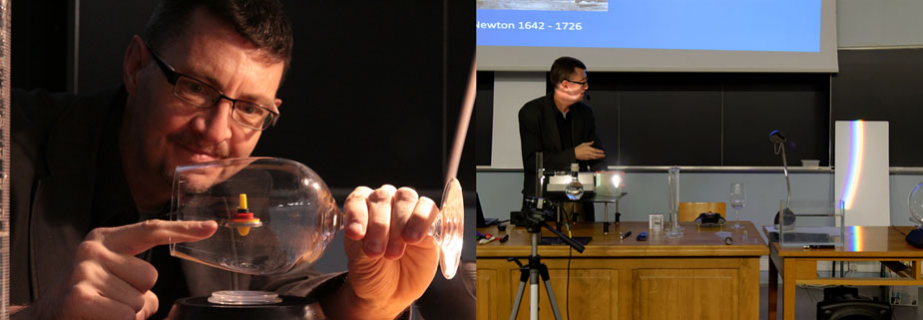
Prof. Dudley at teaching. Credit to: Coraline Lapre.

**Q10: With your years of experience in promoting popular science, can you use a few words to elaborate Rogue waves, supercontinuum and their correlation, to those who does not work in physics or even science?**

A10: The picture we see in the movies of a laser as a powerful beam of light is correct, but lasers are sometimes limited in that they can produce light of one color. However, a wonderful discovery has been that if you focus a laser into a special kind of glass-based material, you can convert the laser into a powerful beam of light that spans all the colors of the rainbow. This is called a supercontinuum, and it finds all sorts of applications - from environmental detection that may help us monitor climate change, to new techniques in medical imaging, and even in the detection of planets around distant stars. Under some conditions, it is even possible to make a very unstable supercontinuum where the behavior of the light mimics the behavior of waves on the ocean! This has been important in allowing us to study how dangerous rogue waves form on the open sea, and also in giving us some ideas about how we might be able to predict them.

